# Ellman’s Assay on the Surface: Thiol Quantification of Human Cofilin-1 Protein through Surface Plasmon Resonance

**DOI:** 10.1021/acs.langmuir.4c02792

**Published:** 2024-09-18

**Authors:** Luiz H. C. Souza, Rayssa G. F. Monteiro, Wellinson G. Guimarães, Ana C. S. Gondim, Eduardo H. S. Sousa, Izaura C. N. Diógenes

**Affiliations:** Departamento de Química Orgânica e Inorgânica, Universidade Federal do Ceará, 60455-760 Fortaleza, CE, Brasil

## Abstract

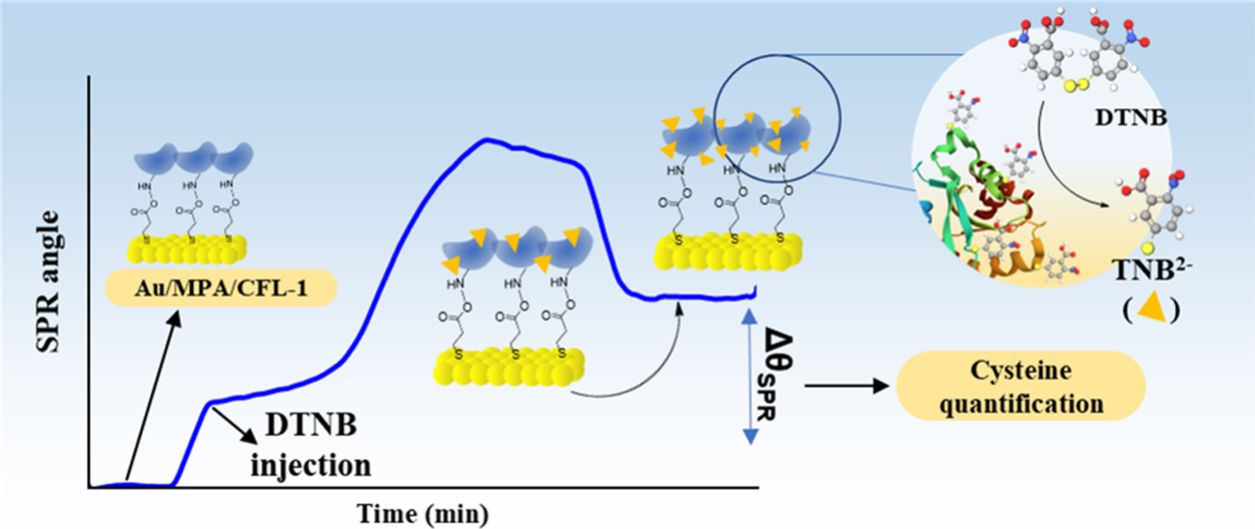

Oxidative stress on cysteine (Cys)-containing proteins has been associated with physiological disorders, as suggested for the human cofilin-1 (CFL-1) protein, in which the oxidized residues are likely implicated in the aggregation process of α-synuclein, leading to severe neuronal injuries. Considering the relevance of the oxidation state of cysteine, quantification of thiols may offer a guide for the development of effective therapies. This work presents, for the very first time, thiol quantification within CFL-1 in solution and on the surface following classic and adapted versions of Ellman’s assay. The 1:1 stoichiometric Ellman’s reaction occurs between 5,5′-dithio-bis(2-nitrobenzoic acid) (DTNB), and the free thiol of the cysteine residue, producing two 2-nitro-5-thiobenzoate (TNB^2–^) ions, one of which is released into the medium. While in solution, the thiol concentration was determined by the absorbance of the released TNB^2–^, on the surface, the mass of the attached TNB^2–^ ion to the protein allowed the quantification by means of the multiparametric surface plasmon resonance (MP-SPR) technique. The SPR angle change after the interaction of DTNB with immobilized CFL-1 gave a surface coverage of 26.5 pmol cm^–2^ for the TNB^2–^ ions (Γ_TNB2–_). The ratio of this value to the surface coverage of CFL-1, Γ_CFL-1_ = 6.5 ± 0.6 pmol cm^–2^ (also determined by MP-SPR), gave 4.1 as expected for this protein, i.e., CFL-1 contains four Cys residues in its native form (reduced state). A control experiment with adsorbed oxidized protein showed no SPR angle change, thus proving the reliability of adapting Ellman’s assay to the surface using the MP-SPR technique. The results presented in this work provide evidence of the heterogenization of Ellman’s assay, offering a novel perspective for studying thiol-containing species within proteins. This may be particularly useful to ensure further studies on drug-like molecules that can be carried out with validated oxidized or reduced CFL-1 or other analogous systems.

## Introduction

Exacerbated oxidative stress has been recognized as an important player in triggering cellular responses leading to a myriad of diseases.^1−4^ In cysteine-containing proteins, oxidative stress operates mainly on cysteine (Cys) residues, resulting in post-translational modifications linked, for instance, to neurodegenerative disorders, as suggested for the human cofilin-1 (CFL-1) protein.^5−7^ CFL-1 is an actin-dynamizing protein present in nonmuscle tissues that possess four Cys residues in its native form (Cys 39, 80, 139, and 147). The major physiological function of CFL-1 is associated with the regulation of cytoskeletal dynamics. Several investigations have revealed the impact of oxidizing the Cys residues in CFL-1 on its physiological function^3,4,8,9^ being likely implicated in the aggregation process of α-synuclein that induces severe neuronal injuries.^4,7^ Considering that cysteine oxidation is indeed related to degenerative disorders, quantifying thiols may offer a guide for the development of effective therapies.

Lately, the surface plasmon resonance (SPR) technique has proven to be a powerful tool for determining the kinetics and thermodynamic parameters of biomolecular interactions aiming at designing drugs for more efficient treatments.^10^ SPR is an evanescent field-based technique in which an evanescent wave propagates along the interface between a sample and a plasmonic metal film. Therefore, any event that occurs at or near the surface of the metal film generates variations in the local refractive index, leading to changes in the SPR angle. In multiparameter SPR (MP-SPR), the detection is improved by a goniometric arrangement scan across a range of refractive indices wider than that of traditional SPR equipment. This feature, in conjunction with the simultaneous use of multiple wavelengths, allows for the measurement of thicker layers and complex systems, such as living cells. The MP-SPR technique represents a great improvement in the measurement of biomolecular interaction parameters, both kinetics and thermodynamics, and is suitable for the detection of small molecules, which is of pivotal importance in the development of new drugs. Furthermore, it is worth noting that the MP-SPR technique is gradually becoming useful for materials science studies, including measuring layer properties like thickness and surface coverage.^11−16^ This ability is highly relevant in designing biosensors, especially plasmonic biosensors since a comprehensive understanding of their properties is necessary to guarantee efficiency, selectivity, orientation, and robustness. In developing plasmonic platforms for sensing purposes, gold is by far the most used metal, not only because of its plasmonic properties but also because of its ease of manipulation, as well as its resistance to oxidation.^11,12^ Furthermore, gold presents a high affinity for sulfur atom leading to the formation of stable and robust self-assembled monolayers (SAMs) that are capable of functionalization.^11,12,17−22^

The quantification of thiol in proteins is usually carried out in solution by following the classical Ellman’s assay.^23−26^ Accordingly, the reaction of the Ellman’s reactant, 5,5′-dithio-bis(2-nitrobenzoic acid) (DTNB), with a free thiol (e.g., cysteine) produces 2-nitro-5-thiobenzoate (TNB^2–^), which strongly absorbs at 412 nm,^25,27^ allowing the spectroscopic determination of the concentration of the thiol species.^23^ Several adaptations of Ellman’s assay have been reported aiming at reducing the limit of detection (LOD) of thiols by means of electronic spectroscopy, chromatography, and electrochemistry,^28−31^ among other techniques. Regarding the electrochemical approaches, which reached LOD as low as 1.17 μmol L^–1^, the use of redox probes is required not only to assess the electron transfer reaction but also to avoid the adsorption of biological fragments on the metallic electrode, leading to the passivation of the surface. To date, however, none of Ellman’s assays, conventional or adapted, have been applied to quantify Cys residues in the CFL-1 protein.

The main goal of this work was to present a method for quantifying the thiol of adsorbed proteins through an adaptation of the well-established Ellman’s assay to the MP-SPR technique using CFL-1 as a model. To this end, we first quantified the Cys residues of CFL-1 in solution by applying classical Ellman’s assay. Having proved the applicability of the conventional method to CFL-1, adaptation to the surface was carried out by applying Ellman’s reaction to the protein immobilized on a functionalized SAM of 3-mercaptopropionic acid (MPA) on gold.

## Materials and Methods

### Chemicals

KCl (99%), KF (99%), NaCl (99%), KOH (90%), suprapur H_2_SO_4_, KH_2_PO_4_ (98%), K_2_HPO_4_ (99%), *N*-(3-(dimethylamino)propyl)-*N*′-ethylcarbodiimide (99%), *N*-hydroxysuccinimide (97%), taurine (99%), l-cysteine (97%), and 3-mercaptopropionic acid (99%), all from Sigma-Aldrich, were used as received. K_4_[Fe(CN)_6_] (98.5%), K_3_[Fe(CN)_6_] (99%), from Acros Organics, 5,5′-dithio-bis(2-nitrobenzoic acid) (Ellman’s reagent, >98%), from Termofischer, tris(2-carboxyethyl)phosphine (97%), from Ambeed, NaOCl (1.6 mol L^–1^), from NEON, and *N*-(2-hydroxyethyl)piperazine-*N*′-2-ethanesulfonic acid (HEPES, 99%), from Serva, were used without further purification.

Phosphate buffer solution (PBS) for electrochemical and impedimetric experiments was prepared by mixing 0.1 mol L^–1^ solutions of KH_2_PO_4_ and K_2_HPO_4_ giving a final buffered solution of pH 8.0. For the SPR measurements, a 0.01 mol L^–1^ solution of HEPES buffer was used, whose pH was adjusted to 8.0 by adding HCl.

An *Escherichia coli* codon-optimized gene of nontagged human cofilin-1 was purchased (GenScript) as a NdeI-*Kpn*I insert into pET30a. This gene was under a T7 promoter and colony selected by a kanamycin-resistant gene. Briefly, this protein was expressed by inducing cells at OD_600 nm_ = 1.2 with 0.5 mM IPTG for 4 h at 37 °C. The cells were collected, lysed, and precleaned using DE52 preswollen resin (DEAE cellulose). This supernatant was applied to an ion-exchange column (CM Sephadex C-50 resin) and further purified by size-exclusion chromatography (S200 Sephacryl). This process led to the isolation of pure protein in the native form (reduced state), where protein concentration was measured by Bradford method^32^ using bovine serum albumin (BSA) and myoglobin as standards. For the protein oxidation, *N*-chlorotaurine (TauCl) compound was previously synthesized by adding 60 μL of a 1.6 mmol L^–1^ solution of NaOCl (stock solution) to 5 mL of a 20 mmol L^–1^ aqueous solution of taurine, according to a reported procedure.^33,34^ This compound was produced *in situ*, and its final concentration was calculated from its molar absorptivity coefficient at 252 nm, ε = 429 L mol^–1^ cm^–1^.^35^ Protein oxidation was conducted in the presence of TauCl following a previously reported procedure.^36^ Briefly, an aliquot of a CFL-1 solution (2 μmol L^–1^) was incubated at 37 °C for 1 h in a 200 μmol L^–1^ solution of TauCl.

Aqueous solutions were prepared using ultrapure water with a resistance of 18 MΩ cm at 25 °C. All other organic solvents used were comparable to analytical grade.

### Equipment

Electrochemical experiments were conducted using an Autolab PGSTAT 302N potentiostat (Echo Chemie, Utrecht, The Netherlands) controlled by Nova software v. 1.11, equipped with an FRA2 module for impedance data collection. The measurements were performed in a conventional three-electrode electrochemical glass cell with a Teflon cap comprising a spiral-shaped platinum, polycrystalline gold (*A*_Geometric_ = 0.0312 cm^2^, BASi), and an Ag/AgCl/Cl^–^ (in saturated KCl) as auxiliary, working and reference electrodes, respectively. Surface plasmon resonance (SPR) measurements were conducted on an MP-SPR Navi 200 OTSO from ©BioNavis Ltd., featuring dual wavelengths of 670 and 785 nm. All experiments were performed at both wavelengths at 24 °C and a flow rate of 10 μL min^–1^. Absorption electronic spectra in the ultraviolet and visible regions (UV–vis) were acquired by using an Agilent Cary 5000 spectrophotometer.

### Electrodes

Previous to all electrochemical and impedimetric measurements, the polycrystalline gold working electrode was submitted to a cleanness procedure including mechanical, chemical, and electrochemical steps. First, the electrode was immersed in “piranha solution” (3H_2_SO_4_:1H_2_O_2_; Caution: piranha solution is a strong oxidant solution that reacts violently with organic compounds), followed by polishing with 0.05 μm alumina, rinsed and sonicated in water, and, as the last step, cycled in a 0.5 mol L^–1^ solution of suprapur H_2_SO_4_ until the achievement of the typical voltammetric profile of a clean polycrystalline gold surface.^37^ The cyclic voltammogram (CV) of the polycrystalline gold surface allowed the determination of the electroactive area that is essential for normalizing the electrochemical and impedimetric data. For the SPR experiments, gold sensor slides from ©BioNavis Ltd. (SPR102-AU-60) composed of a 50 nm gold layer deposited on a 2 nm layer of chromium were used. The cleanness procedure of these slides included only immersion in the piranha solution followed by an exhaustive water rinse and drying under argon flux.

### Modification of the Gold Surface: from the SAM of MPA to the Immobilization of CFL-1

Two steps of modification preceded the immobilization of the CFL-1 protein on the clean gold surfaces with a few differences depending on whether it was gold polycrystalline electrodes for electrochemical and impedimetric measurements or gold slides for SPR experiments. For the gold polycrystalline electrode, it was first immersed in a 10 mmol L^–1^ aqueous solution of MPA for 12 h, according to the procedure reported in the literature,^38^ to produce a SAM of MPA on gold (Au/MPA). Second, the activation of the carboxylic groups of the adsorbed MPA molecules was achieved by following published protocols^38,39^ with minor modifications. Briefly, the produced Au/MPA electrode was immersed for 30 min in an aqueous solution containing 0.05 mol L^–1^ of *N*-(3-(dimethylamino)propyl)-*N*′-ethylcarbodiimide hydrochloride (EDC) and 0.03 mol L^–1^ of *N*-hydroxysuccinimide (NHS) leading to the production of Au/MPA/EDC:NHS, a surface able to form amide bonds with the CFL-1 protein. The covalent immobilization of CFL-1 was achieved by immersing the Au/MPA/EDC:NHS electrode in a 0.1 mol L^–1^ solution of PBS (pH 8.0) containing 1.0 μmol L^–1^ of CFL-1 at 24 °C. Keeping constant the concentration and temperature, different immersion times were studied to ensure an optimum amount of the immobilized protein.

For the gold slide sensors (SPR experiments), the first step, i.e., the formation of the MPA SAM, was essentially the same as that followed for the electrochemical and impedimetric measurements. The produced Au/MPA surface was then placed at the equipment sample holder for the injection (10 μL min^–1^, 24 °C) of a 10.0 mmol L^–1^ solution of HEPES buffer up to stabilization of the SPR angle (θ_SPR_). After that, EDC and NHS (same concentration mentioned above) in HEPES buffer were injected for *in situ* activation of the carboxylic groups of MPA. Upon signal stabilization and washing with HEPES, a 1 μmol L^–1^ solution of CFL-1 in HEPES buffer (10.0 mmol L^–1^) was injected for monitoring the protein immobilization. For the SPR experiments with the oxidized protein, the same experimental protocol was followed but using CFL-1 after incubation for 1 h in a 200 μmol L^–1^ solution of TauCl, as previously detailed for the procedure of the protein oxidation.

### Quantification of the Adsorbed Material (Surface Coverage)

The surface coverages (Γ, in mol cm^–2^) on the gold surfaces were calculated by SPR applying the Feijter method,^40^ which considers the increment in the refractive index and the optical thickness of the layer as given by eq 1:
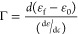
1where ε_f_ and ε_0_ are, respectively, the refractive indexes of the layer and the medium, and dε/d*c* is the specific increment of the refractive index. The optical thickness, *d*(ε_f_ – ε_0_), in turn, is obtained from the SPR angle change (Δθ_SPR_) as follows:
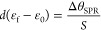
2where *S* is an equipment constant (1.10 and 0.61 for lasers at 670 and 785 nm, respectively). Substituting eq 2 into 1, we obtain eq 3, i.e., a linear relation between Γ and Δθ_SPR_:
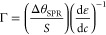
3To avoid error in data interpretation regarding the Δθ_SPR_ response of small molecules, a correction^41^ was added to calculate  for determining Γ of 2-nitro-5-thiobenzoate (TNB):
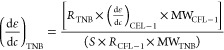
4where *R* is the experimental SPR response at saturation, *S* is the stoichiometric ratio, and MW is the molecular weight.

The thickness of the adsorbed layers were determined by using the Layer Solver Software (©Bionavis) assuming a refractive index of 1.33 for the HEPES solution and 1.52 for CFL-1 based on typically reported values.^42^

### Ellman’s Assays

Ellman’s assays were performed in solution and on the surface to determine the amount of cysteine residues in the CFL-1 protein in the reduced (native) and oxidized (control) states. Following the standard protocol,^24,26^ it was assumed, in both solution and on surface, a 1:1 stoichiometric reaction between 5,5′-dithio-bis(2-nitrobenzoic acid) (DTNB) and the free thiol group of each of the four cysteine residues of CFL-1.

In solution, the reaction of DTNB with cysteine gives 2-nitro-5-thiobenzoate (TNB^2–^), which strongly absorbs at 412 nm,^25,27^ allowing the spectroscopic determination of the cysteine residue concentration in CFL-1 by following the molar absorptivity of the TNB^2–^ ion.^23^ A calibration curve of the absorbance change at 412 nm (ΔAbs_412_) versus cysteine concentration ([Cys]) was constructed with the data collected during the titration of a DTNB solution with free cysteine (Figure S1). For Ellman’s assay, the UV–vis spectra were acquired in a 0.01 mol L^–1^ stock solution of DTNB in 0.1 mol L^–1^ PBS (pH 8.0). The experiments were conducted for the protein in the native (1.73 μmol L^–1^) and oxidized (1.53 μmol L^–1^) states with a final DTNB concentration of 200 μmol L^–1^. The UV–vis spectra were recorded during at least 30 min until no change was seen at 412 nm. All spectroscopic data obtained for CFL-1 was applied to the calibration curve (Figure S1) for calculating the concentration of the Cys residues within the protein.

For the assays on the surface, it was considered the Δθ_SPR_ parameter to indirectly quantify the amount of cysteine residues in the CFL-1 protein immobilized on gold (Au/MPA/EDC:NHS/CFL-1). The experiments were recorded by injecting a 10 mmol L^–1^ solution of HEPES containing 200 μmol L^–1^ DTNB at a flow rate of 10 μL min^–1^ on Au/MPA/EDC:NHS/CFL-1. After signal stabilization and washing with HEPES, the obtained Δθ_SPR_ values were treated with the Feijter method for calculating the amount of TNB^2–^ ions immobilized within the protein. A control experiment was run with the oxidized protein (CFL-1ox) to guarantee that the change in the SPR angle upon DTNB injection was due to TNB^2–^ as a consequence of the interaction of the cysteine residues of CFL-1 with DTNB.

## Results and Discussion

### Immobilization of CFL-1 on the MPA SAM

**Figure 1:**
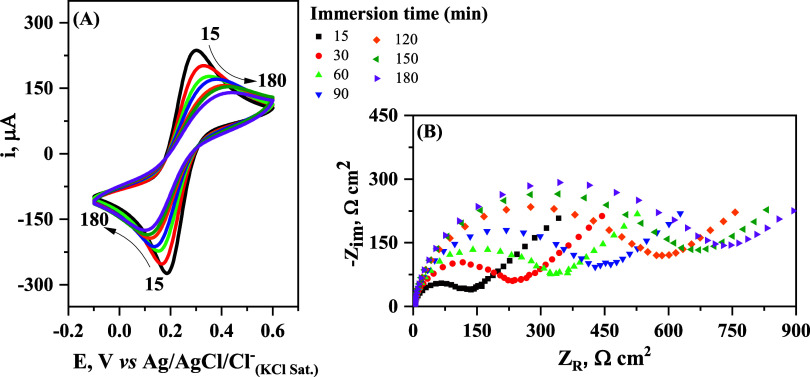
(A) Cyclic voltammograms at 100 mV s^–1^ and (B) Nyquist diagrams of Au/MPA/EDC:NHS after different immersion times in 1.0 μmol L^–1^ CFL-1 in PBS (0.1 mol L^–1^, pH 8.0). Measurements were conducted in a 0.5 mol L^–1^ solution of KF containing 2.5 mmol L^–1^ [Fe(CN)_6_]^4–/3–^ at 24 °C and pH ∼ 6.0.

Previously to the protein immobilization, the gold electrode was spontaneously modified with 3-mercaptopropionic acid (MPA), resulting in a self-assembled monolayer (SAM), Au/MPA, which was characterized by cyclic voltammetry (CV) and electrochemical impedance spectroscopy (EIS) in a solution containing [Fe(CN)_6_]^3–/4–^ (Figure S2). The values of fractional coverage (θ) and charge transfer resistance (*R*_CT_) for Au/MPA were determined as 0.88 and 41.11 Ω cm^2^, respectively (Figure S2 and Table S1). After this first step, the Au/MPA electrode was immersed in a solution containing EDC and NHS to activate the carboxylic groups^38,39^ of MPA forming the Au/MPA/EDC:NHS surface. This surface, in turn, was immersed in a buffer solution (pH 8.0) containing the CFL-1 protein at different times. The entire process of self-assembly modification was monitored by CV and EIS. Figure 1 shows the cyclic voltammograms and Nyquist diagrams obtained for Au/MPA/EDC:NHS after different immersion times in a 0.1 mol L^–1^ solution of PBS (pH 8.0) containing 1.0 μmol L^–1^ of CFL-1.

Table 1 summarizes the dependence of the electrochemical and impedimetric data on the immersion time of Au/MPA/EDC:NHS in CFL-1 solution, as obtained from the voltammetric curves and Nyquist diagrams shown in Figures 1 and S2.

**Table 1:** Values of Charge Transfer Resistance (*R*_CT_), Fractional Coverage (θ), and Peak Potential Separation (Δ*E*_p_) Obtained for Au/MPA/EDC:NHS after Different Immersion Times in Solution Containing CFL-1^a^

immersion time in CFL-1 solution (min)	*R*_CT_ (10^2^ Ω cm^2^)	θ	Δ*E*_p_ (mV vs Ag/AgCl)
0	0.23	0.78	78.0
15	1.27	0.960	110.0
30	2.47	0.979	140.0
60	3.04	0.983	220.0
90	4.06	0.988	234.0
120	5.68	0.991	307.0
150	6.82	0.992	309.0
180	7.02	0.993	313.0

a Data were collected from CV and EIS measurements (Figures 1 and S2) in a 0.5 mol L^–1^ solution of KF containing 2.5 mmol L–1 of [Fe(CN)_6_]^4–/3–^.

The impedimetric and electrochemical data obtained for the redox probe complex [Fe(CN)_6_]^4–/3–^ during the modification steps of the gold surface clearly indicate that the electrode is progressively passivated, with the most abrupt change being observed at the final step: from 23 Ω cm^2^ for Au/MPA/EDC:NHS to 127 Ω cm^2^ after just 15 min of immersion in solution containing CFL-1. As can be ascertained from the CV and EIS experiments, the blockage effect of CFL-1 is shown to be time-dependent (see Table 1), reaching a plateau after 2 h of immersion. Indeed, the values of *R*_CT_ and Δ*E*_p_ increase with the increase of the immersion time due to the difficulty of the redox probe species in assessing the underlying gold surface, where the heterogeneous electron transfer reaction takes place.

**Figure 2:**
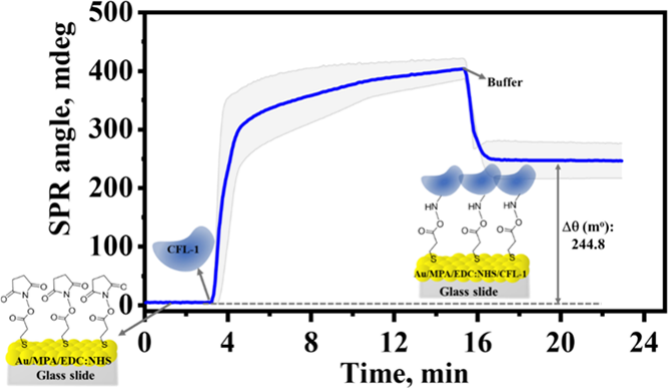
SPR sensorgram obtained for Au/MPA/EDC:NHS in HEPES (10 mmol L^–1^, pH 8.0) flow following the injection of a 1.0 μmol L^–1^ solution of CFL-1 (in HEPES) and buffer rinsing. The dotted gray line indicates the baseline extrapolation, and the shaded gray area refers to the standard deviation from four replicates. All solutions were injected at a flow rate of 10 μL min^–1^ at 24 °C. Laser: 670 nm.

The immobilization process of the CFL-1 protein on Au/MPA/EDC:NHS was also monitored under flow conditions through the MP-SPR technique. All MP-SPR measurements were conducted in 10 mmol L^–1^ solution of HEPES buffer (pH 8.0) rather than PBS because of a better signal-to-noise ratio. Figure 2 shows the obtained sensorgram along with illustrative representations to account for the formation of Au/MPA/EDC:NHS/CFL-1.

After signal stabilization (at ca. 3.5 min), a 1.0 μmol L^–1^ solution of CFL-1 prepared in HEPES buffer was injected giving a strong SPR angle change, which was ascribed to the immobilization of the protein through amide bonds with the activated carboxylic groups of the MPA SAM. A plateau was reached after about 12 min of CFL-1 flow when HEPES buffer was injected for washing and removal of nonbonded proteins. At this point, it is relevant to mention that 12 min is enough to reach a maximum of CFL-1 immobilization through SPR flow conditions, whereas 2 h of immersion is required to reach a plateau when the process occurs via self-assembly (as indicated by CV and EIS, see Table 1). This is an expected result, indeed, since the flow conditions of the SPR experiments favor the mass transport, accelerating the immobilization of the protein.^43^

An SPR angle change of 244.8 mdeg (mean value, see Table S2) from the baseline (prior to the CFL-1 injection) was applied to the Feijter method (eqs 1–3), giving a surface coverage (Γ) of 6.5 ± 0.6 pmol cm^–2^. The SPR data (intensities and angle) obtained at two different wavelengths (670 and 785 nm), Figure S3, were analyzed using Layer Solver software (Bionavis), providing a film thickness of 1.53 nm for the protein. Notably, this dimension is not far from the expected radius for a completely globular protein of molecular weight within 10–20 kDa (*R*_min_ = 1.42–1.78 nm),^44^ supporting a monolayer of monomers of CFL-1 was anchored. In addition, our findings are consistent with a film thickness of 1.43 nm reported for the adsorption of bovine serum albumin on a SAM of 11-mercapto-1-undecanol.^45^

### Ellman’s Assay

#### Ellman’s Assay in Solution

**Figure 3:**
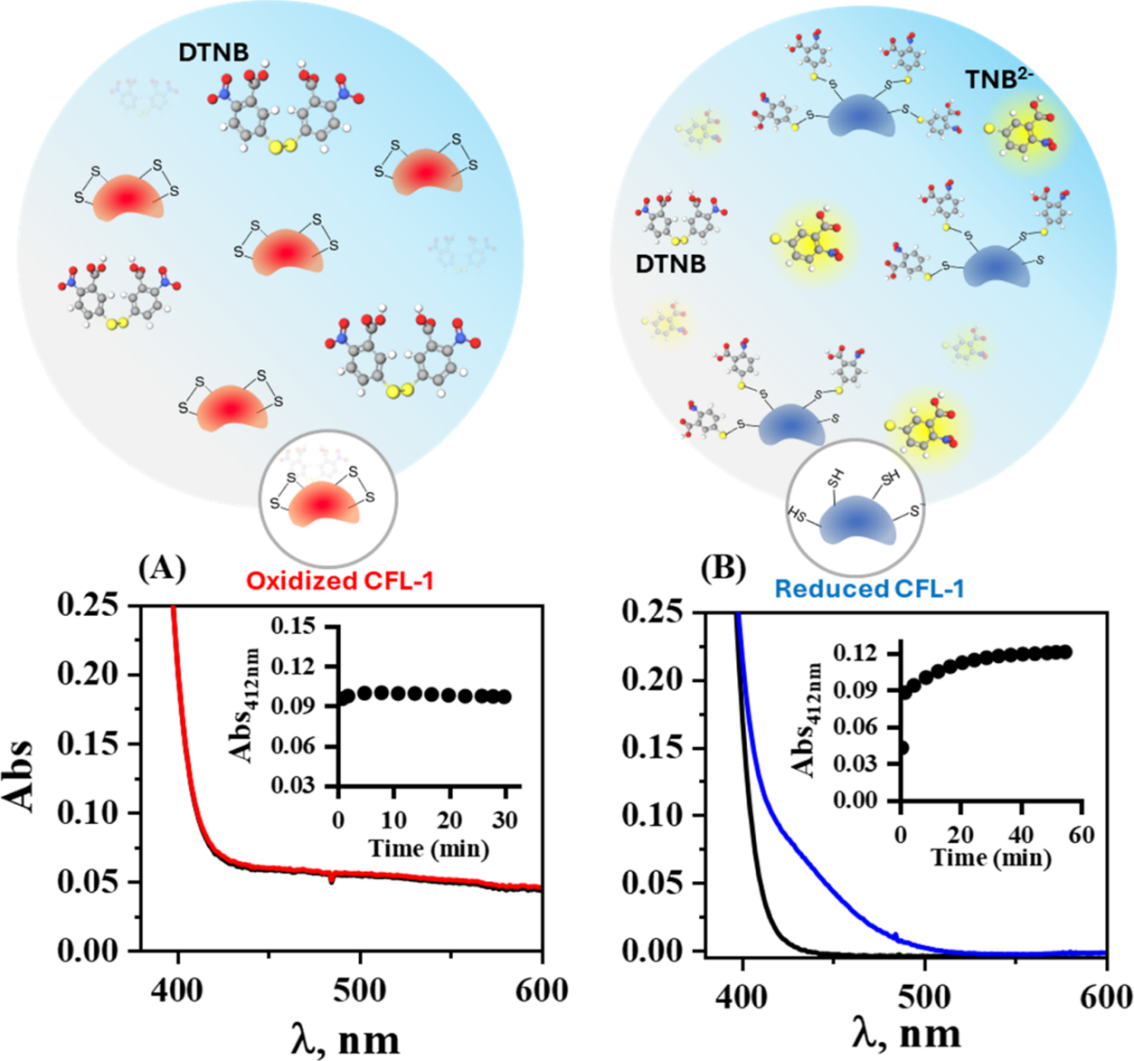
Top: cartoon illustration showing the noninteraction of DTNB with oxidized CFL-1 and the reaction of DTNB with thiol groups of the reduced CFL-1 producing TNB^2–^. Bottom: UV–vis spectra obtained for the CFL-1 protein in (A) oxidized (1.53 μmol L^–1^, red line) and (B) native (1.73 μmol L^–1^, blue line) states in 0.1 mol L^–1^ solution of PBS (pH 8.0) after the addition of DTNB (200 μmol L^–1^), *t* = 30 min (A) and 60 min (B). Black lines refer to the spectra of a 200 μmol L^–1^ solution of DTNB without protein. Insets: curves of absorbance at 412 nm as a function of the interaction time with DTNB.

Prior to the determination of the amount of cysteine within CFL-1 adsorbed on gold, Ellman’s assay was performed for this protein in solution since to date we could not find any report on such specific measurement. According to the standard protocol,^24,26^ a 1:1 stoichiometric reaction between DTNB and cysteine produces two TNB^2–^ ions, one of which is bonded to the thiol group of the protein and the other is released to the solution. The released ion strongly absorbs at 412 nm,^25,27^ thus allowing the thiol spectroscopic determination. Following this approach, the cysteine concentration in CFL-1 might be estimated by following the molar absorptivity of the TNB^2–^ ion.^23^ Accordingly, a total of four TNB^2–^ ions should be produced upon the reaction of DTNB with the free thiol group of each of the four cysteine residues of CFL-1. Figure 3 illustrates the UV–vis spectra obtained for native (reduced) and oxidized (control) CFL-1 in the presence of DTNB. We also included a cartoon illustration to facilitate a visual comprehension of the interaction between DTNB and CFL-1.

As can be seen in Figure 3A, there is no absorbance change at 412 nm, indicating all cysteine residues of CFL-1 were oxidized upon the reaction with *N*-chlorotaurine (TauCl). On the other hand, the spectra obtained for the native protein, Figure 3B, exhibit a meaningful absorbance change at ca. 412 nm after reaction with DTNB. Correlating the value of the absorbance maximum in Figure 3B with the experimentally determined molar absorptivity coefficient of TNB^2–^ (12,437.0 L mol^–1^ cm^–1^, Figure S1), we estimated the concentration of this ion in the sample as 5.53 μmol L^–1^. Following the Ellman’s approach, a value of 3.19 was found for the TNB^2–^/CFL-1 ratio, meaning that ca. 80% of the CFL-1 cysteine residues of the native CFL-1 protein are in the reduced state (thiol form). Cysteine reactivity and accessibility may be an issue for quantitative measurements or modification,^46^ which was previously reported for DTNB with certain proteins. In those cases, cysteine residues were underestimated,^47−49^ which seems to be the case here as well. In addition, Ellman’s assay has a limit of detection of ca. 3 μmol L^–1^ being not sensitive enough for low-level detection,^50^ which is the case in this study.

#### Ellman’s Assay on the Surface

**Figure 4:**
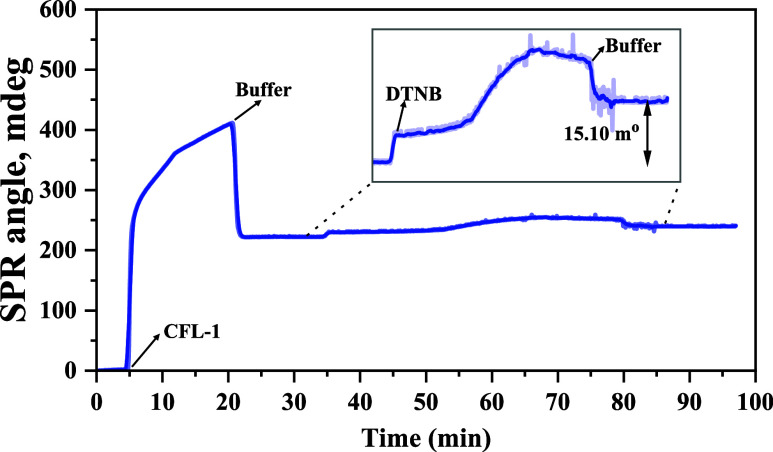
SPR sensorgram obtained for Au/MPA/EDC:NHS in HEPES buffer (10 mmol L^–1^, pH 8.0) during injections of CFL-1 (1 μmol L^–1^) and DTNB (200 μmol L^–1^). CFL-1 and DTNB solutions were prepared in 10 mmol of L^–1^ HEPES (pH 8.0). All solutions were injected at a flow rate of 10 μL min^–1^ at 24 °C. Laser: 670 nm.

On the surface, the reaction of DTNB with the cysteine residues of CFL-1 proceeds in the very same way as in solution. In this case, however, the response signal for the cysteine quantification is based on the mass increment resultant from the TNB^2–^ ions attached to the protein (one ion per Cys). For detecting such mass increment, the variation of the SPR angle was applied to the Feijter method^40^ to determine the surface coverage of TNB^2–^. Figure 4 shows the SPR sensorgram obtained for Au/MPA/EDC:NHS during the CFL-1 immobilization followed by the injection of DTNB. This sensorgram is the result of subtracting the sensorgram obtained up to the formation of Au/MPA/EDC:NHS/CFL-1 (Figure 2) from that acquired in the reference channel when only the DTNB solution was injected over Au/MPA/EDC:NHS (Figure S4). This control sensorgram confirmed DTNB does not interact with the modified surface.

After signal stabilization, a 200 μmol L^–1^ solution of DTNB was injected for ca. 40 min followed by washing with HEPES buffer, giving a Δθ_SPR_ of 15.1 m^o^. Assuming the DTNB is enough to saturate all binding sites of the immobilized CFL-1 protein, the Feijter equation (eq 3) was used for determining the surface coverage of TNB^2–^ (Γ_TNB^2–^_) as 26.5 pmol cm^–2^ (mean value). Table S2 summarizes the values of mass and surface coverage obtained from the replicates conducted for MP-SPR data acquisition. A value of 4.1 ± 0.5 (see Table S2) was found for the Γ_TNB_^2–^/Γ_CFL-1_ ratio, which is consistent with the expected stoichiometric ratio (4TNB^2–^:1CFL-1). This result hints that the CFL-1 protein is immobilized on the surface in such a conformation that retains the four cysteine residues available for reacting with DTNB. The immobilization of CFL-1 onto gold surface seems to facilitate DTNB reaction illustrating the importance of the microenvironment for the reactivity of DTNB.^49^

**Figure 5:**
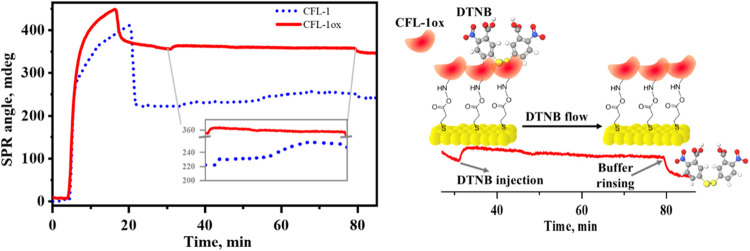
Left: SPR sensorgrams obtained for Au/MPA/EDC:NHS in HEPES buffer (10 mmol L^–1^, pH 8.0) during injections of oxidized (CFL-1ox, red solid line) and native (CFL-1, short-dotted blue line) proteins, and DTNB (200 μmol L^–1^). CFL-1 and DTNB solutions were prepared in 10 mmol L^–1^ HEPES (pH 8.0). All solutions were injected at a flow rate of 10 μL min^–1^ at 24 °C. Laser: 670 nm. Inset: zoomed-in view of the sensorgram during DTNB injection. Right: schematic illustration of the injection of DTNB onto the immobilized oxidized CFL-1 resulting in no interaction and release after buffer rinsing.

To validate the conclusion that the signal variation observed in the SPR sensorgram shown in Figure 4 was indeed a result of the binding of TNB^2–^ to the cysteine residues of CFL-1, an SPR control experiment was conducted for the immobilized oxidized protein (CFL-1ox). For this experiment, a solution of CFL-1ox in HEPES buffer (10 mmol L^–1^, pH 8.0) was injected over Au/MPA/EDC:NHS followed by buffer rinsing and injection of a 200 μmol L^–1^ solution of DTNB. The SPR sensorgram obtained for CFL-1ox (red solid line) is shown in Figure 5 along with the one acquired for the native protein (short-dotted blue line) for comparative purpose.

On the contrary of what is observed in the sensorgram obtained for the immobilized native protein (short-dotted blue line in Figure 5), the curve obtained for Au/MPA/EDC:NHS/CFL-1ox (red solid line) showed no net SPR angle change after DTNB injection. In fact, the reaction of CFL-1 with TauCl leads to the oxidation of cysteine residues to cystine, thus giving oxidized protein that no longer has free thiol groups available for interaction with DTNB, as schematically shown in Figure 5. Therefore, the lack of SPR angle variation upon DTNB injection over Au/MPA/EDC:NHS/CFL-1ox indicates not only that the immobilized protein is fully oxidized but also that the SPR angle change seen in Figure 4 must be due to the attachment of TNB^2–^ to the native CFL-1 protein.

## Conclusions

In this study, we quantified, for the very first time, the cysteine residues within the human CFL-1 protein in solution and on the surface following, respectively, the conventional and adapted versions of Ellman’s assay. In these approaches, the amount of cysteine was either indirectly or directly determined by the product (TNB^2–^) of the 1:1 stoichiometric reaction between Ellman’s reactant (DTNB) and the free thiol of the cysteine residues within CFL-1 protein in solution and on the surface, respectively. In solution, the reaction was followed by the strong absorption of TNB^2–^ while on the surface, the mass increment of the attached TNB^2–^ ion to the protein was detected by MP-SPR. In the latter condition, gold surfaces were spontaneously modified with 3-mercaptopropionic acid (MPA), followed by activation with EDC and NHS (Au/MPA/EDC:NHS) to allow the immobilization of the protein through amide bonds. MP-SPR was used to follow the real-time immobilization of CFL-1 producing Au/MPA/EDC:NHS/CFL-1 and to quantify the cysteine residues. Applying the Feijter equation, a surface coverage (Γ_CFL-1_) of 6.5 ± 0.6 pmol cm^–2^ was found for CFL-1 over the Au/MPA/EDC:NHS surface with a film thickness of 1.53 nm. Upon injection of DTNB over Au/MPA/EDC:NHS/CFL-1, an SPR angle change of 15.1 m^o^ was observed, indicating a surface coverage of 26.5 pmol cm^–2^ for the TNB^2–^ ions (Γ_TNB_^2–^), giving a Γ_TNB_^2–^/Γ_CFL-1_ ratio of 4.1 ± 0.5, a result that is in close agreement with the expected value of four TNB^2–^ ions for one CFL-1 protein. Control experiments with the adsorbed oxidized protein proved the reliability of adapting Ellman’s assay to the surface using MP-SPR, thus offering a novel perspective for thiol quantification within proteins.

Given the significance of cysteine residues in the biological activity of various proteins such as CFL-1, it is advantageous to possess a technique for assessing the integrity of these residues while studying their molecular function. The oxidation of cysteine residues in CFL-1 has the capacity to impact neurological disorders by modulating several processes, including protein aggregation. Hence, it is critical to verify the presence of those species on the surface when an SPR apparatus is used to investigate small molecules that are selective to oxidized or reduced versions of CFL-1. By utilization of this approach in future studies, it is feasible to pinpoint particular potential drug candidates.

## Supporting Information Available

Complementary experimental details and results, including UV–vis spectra, Nyquist diagrams, and SPR curves (PDF)
