# Supplementary material for: Ellman’s Assay on the Surface: Thiol Quantification of Human Cofilin-1 Protein through Surface Plasmon Resonance
Source: Langmuir. 2024 Sep 18;40(39):20707–14. doi: 10.1021/acs.langmuir.4c02792 (PMC11447915; doi:10.1021/acs.langmuir.4c02792)
Supplement: Supplementary file 1 — la4c02792_si_001.pdf [file la4c02792_si_001.pdf]

Ellman's Assay on Surface: Thiol Quantification of Human Cofilin-1 Protein Through Surface Plasmon Resonance

Luiz H. C. Souza, Rayssa G. F. Monteiro, Wellinson G. Guimarães, Ana C. S. Gondim, Eduardo H. S. Sousa, Izaura C. N. Diógenes*

*izaura@dqoi.ufc.br

Departamento de Química Orgânica e Inorgânica, Universidade Federal do Ceará, 60455-760, Fortaleza-CE, Brasil

Supporting information

Molar absorptivity coefficient of 2-nitro-5-thiobenzoate (TNB^{2-}) in cysteine solution

To determine the concentration of cysteine in CFL-1 following the standard Ellman's assay, the molar absorptivity coefficient of TNB^{2-} was first calculated (12437 L mol^{-1} cm^{-1}) by titrating a DTNB solution with different concentrations of cysteine. Figure S1 shows the obtained UV-Vis spectra and the plot of the absorbance at 412 nm as function of the cysteine concentration, [Cys].

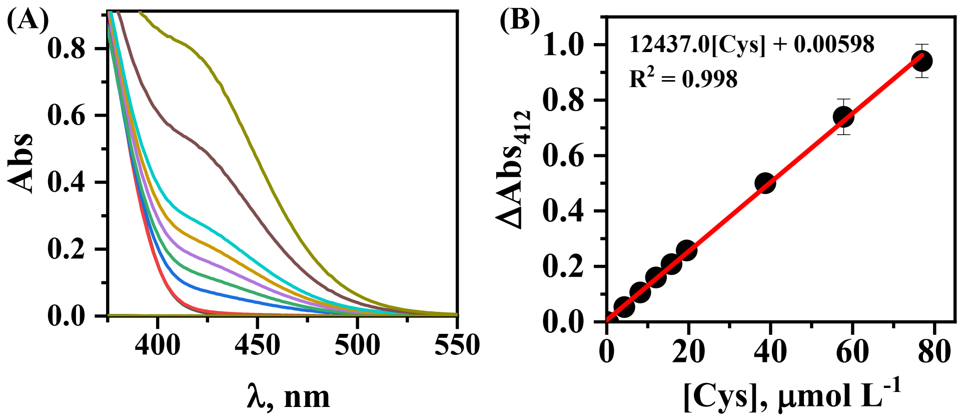

Figure S1. (A) UV-Vis spectra of a 200 µmol L^{-1} solution of DTNB after successive additions of Cys (0 - 80 µmol L^{-1}), (B) plot of the absorbance at 412 nm as a function of the cysteine concentration, [Cys].

Electrochemical and impedimetric results

Figure S2 shows the cyclic voltammograms and Nyquist diagrams obtained in KF solution containing the redox probe $[Fe(CN)_6]^{3-/4-}$ after each modification step of a gold polycrystalline electrode. At first, the gold electrode was immersed in a 10 mmol L^{-1} solution of 3-mercaptopropionic acid (MPA) for 12 h giving the modified electrode Au/MPA. This modified surface was immersed in an aqueous solution containing 0.05 mol L^{-1} of *N*-(3-dimethylaminopropyl)-*N*′-ethylcarbodiimide hydrochloride (EDC) and 0.03 mol L^{-1} of *N*-hydroxysuccinimide (NHS) for activation of the MPA carboxylic groups thus producing Au/MPA/EDC:NHS. In the final step, the Au/MPA/EDC:NHS electrode was immersed for 3 h in a 0.1 mol L^{-1} phosphate buffer solution (pH 8.0) containing 1.0 µmol L^{-1} of CFL-1. Table S1 summarizes the impedimetric and voltammetric data during the modification procedure.

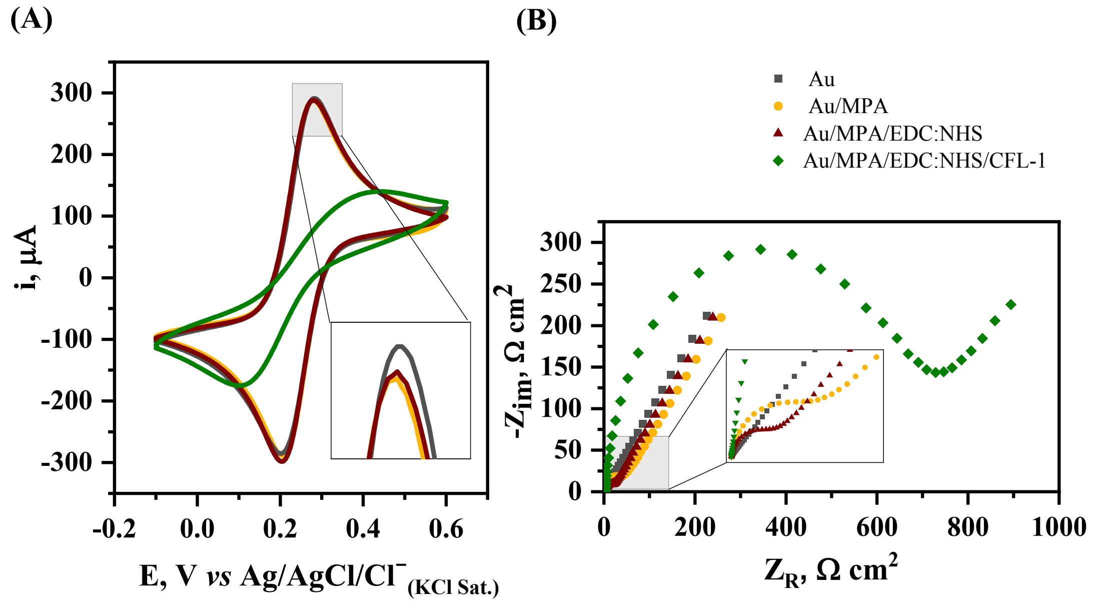

Figure S2. (A) Cyclic voltammograms at 100 mV s^{-1} and (B) Nyquist diagrams of bare gold electrode (Au) (■), Au/MPA (●), Au/MPA/EDC:NHS (▲), and Au/MPA/EDC:NHS/CFL-1 (♦). Measurements were conducted in a 0.5 mol L^{-1} solution of KF (pH ~6.0) containing 2.5 mmol L^{-1} of $[Fe(CN)_6]^{4-/3-}$ at 24°C.

Table S1. Values of charge transfer resistance (R_{CT}), fractional coverage (θ), and peak potential separation (ΔEp) obtained for bare gold (Au) and for the modified surfaces Au/MPA, Au/MPA/EDC:NHS and Au/MPA/EDC:NHS/CFL-1. Data were collected from CV and EIS curves shown in Figure S2.

Surface	R_{CT} (Ω cm^2)	θ	ΔEp (mV vs Ag/AgCl)
Au	5.0	--	78.0
Au/MPA	41.1	0.88	73.0
Au/MPA/EDC:NHS	23.0	0.78	78.0
Au/MPA/EDC:NHS/CFL-1*	702.0	0.993	313.0

*Produced upon immersion of Au/MPA/EDC:NHS for 3 h in a 0.1 mol L^{-1} solution of PBS (pH 8.0) containing 1 μmol L^{-1} of CFL-1.

Surface coverage of CFL-1 and TNB^{2-}

The surface coverage of the TNB^{2-} ions ($\Gamma_{TNB}{}^{2-}$) was determined by MP-SPR to allow the indirect quantification of the cysteine residues within the immobilized CFL-1 protein. Table S2 presents the four replicate data obtained by MP-SPR for mass and surface coverage determination of CFL-1 and TNB^{2-}.

Table S2. Values of SPR angle change ($\Delta\theta_{SPR}$), mass (m) and surface coverage (Γ) of CFL-1 and TNB^{2-}, and $\Gamma_{TNB}{}^{2-}$ / $\Gamma_{CFL\text{-}1}$ ratio as obtained by MP-SPR using the Feijter model. Mean value ($\bar{\chi}$) and confidence intervals (CI) of all measurements are given at the bottom of the table.

	CFL-1			TNB^{2-}			
	$\Delta\theta_{SPR}$, mdeg	m (ng cm^{-2})	$\Gamma_{CFL\text{-}1}$ (pmol cm^{-2})	$\Delta\theta_{SPR}$, mdeg	m (ng cm^{-2})	$\Gamma_{TNB}{}^{2-}$ (pmol cm^{-2})	$\frac{\Gamma_{TNB^{2-}}}{\Gamma_{CFL-1}}$
	224.4	110.0	5.9	15.1	5.0	25.6	4.3
	265.1	129.9	7.0	16.2	5.4	27.5	3.9
	222.5	109.0	5.9	11.9	3.9	19.6	3.3
	269.1	131.9	7.1	19.8	6.6	33.6	4.7
$\bar{\chi}$:	244.8	119.9	6.5	15.7	5.2	26.5	4.1
*CI:	21.9	±10.7	±0.6	2.9	±1.0	±5.0	±0.5

*$CI = \bar{\chi} \pm z\frac{s}{\sqrt{n}}$, where $\bar{\chi}$ is the sample mean, z is the confidence level, s is the standard deviation, and n is the sample size.

Thickness of the adsorbed layers

The thickness of the layer comprising of MPA, EDC:NHS, and CFL-1 before and after interaction with DTNB was determined by MP-SPR using laser beams of two different wavelengths. Figure S3 shows the plots of the SPR intensity as function of SPR angle obtained for Au/MPA/EDC:NHS/CFL-1 at wavelengths of 670 and 785 nm before and after interaction with DTNB.

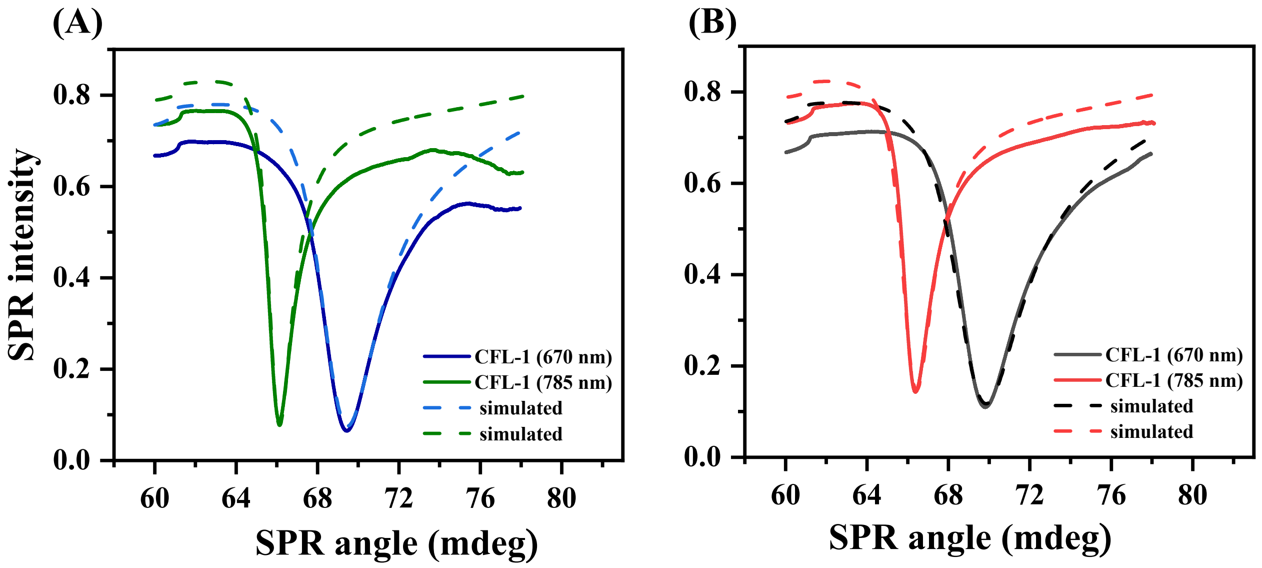

Figure S3. Experimental (solid lines) and simulated (dashed lines) plots of SPR intensity as function of SPR angle for Au/MPA/EDC:NHS/CFL-1 before (A) and after (B) interaction with DTNB at wavelengths of 670 and 785 nm. Layer Solver software (Bionavis®) was used for data simulation.

The data shown in Figure S3 (A) were treated with the Layer Solver software (Bionavis®) giving a thickness of 1.53 nm.

Reference SPR sensorgram

Figure S4 shows the sensorgrams obtained during injections of CFL-1 and DTNB including that obtained in the reference channel when only DTNB was injected over Au/MPA/EDC:NHS.

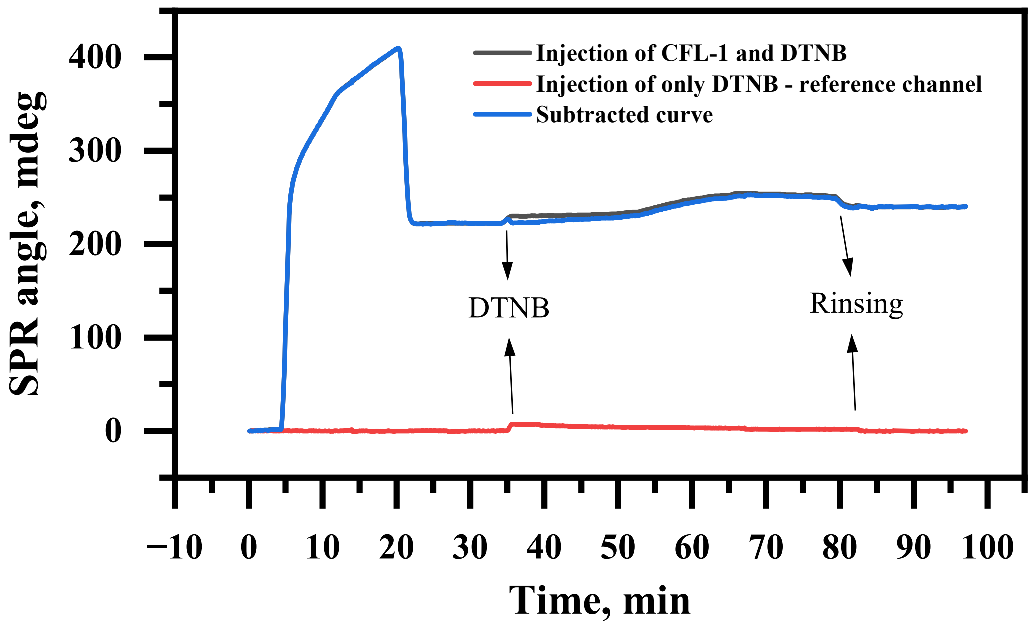

Figure S4. SPR sensorgrams obtained for Au/MPA/EDC:NHS in HEPES buffer (10 mmol L^{-1}, pH 8.0) during injections of CFL-1 (1 µmol L^{-1}) and DTNB (200 µmol L^{-1}) before (black line) and after (blue line) the subtraction of the reference curve (red line). The reference curve was obtained for Au/MPA/EDC:NHS during injection of DTNB (200 µmol L^{-1}) without the protein. CFL-1 and DTNB solutions were prepared in 10 mmol L^{-1} HEPES (pH 8.0). All solutions were injected at a flow rate of 10 µL min^{-1} at 24 °C. Laser: 670 nm.
